# Supplementary figures and images for: The spread of pESI-mediated extended-spectrum cephalosporin resistance in Salmonella serovars—Infantis, Senftenberg, and Alachua isolated from food animal sources in the United States
Source: PLoS One. 2024 Mar 14;19(3):e0299354. doi: 10.1371/journal.pone.0299354 (PMC10939224; doi:10.1371/journal.pone.0299354)

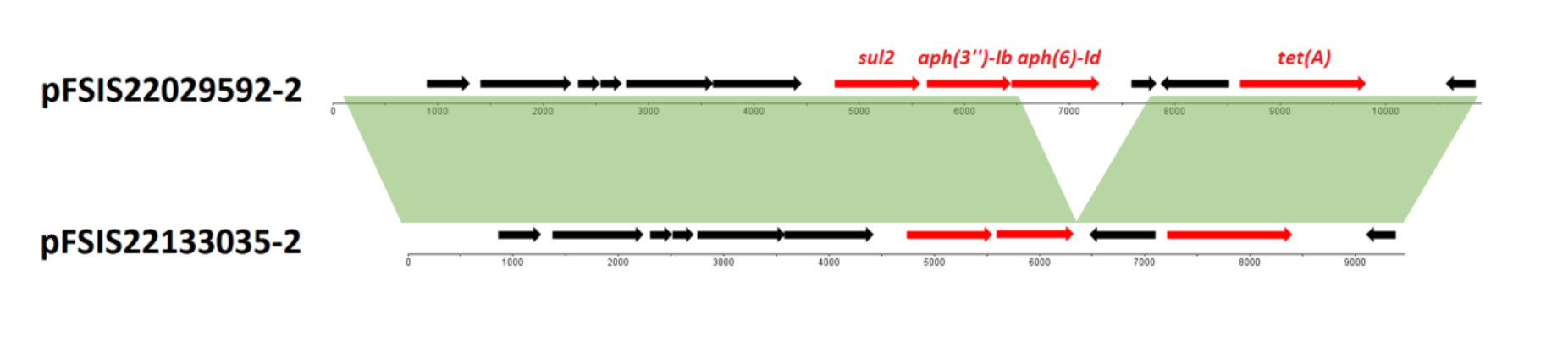

Supplement: S1 Fig — (TIF) [file pone.0299354.s003.tif]

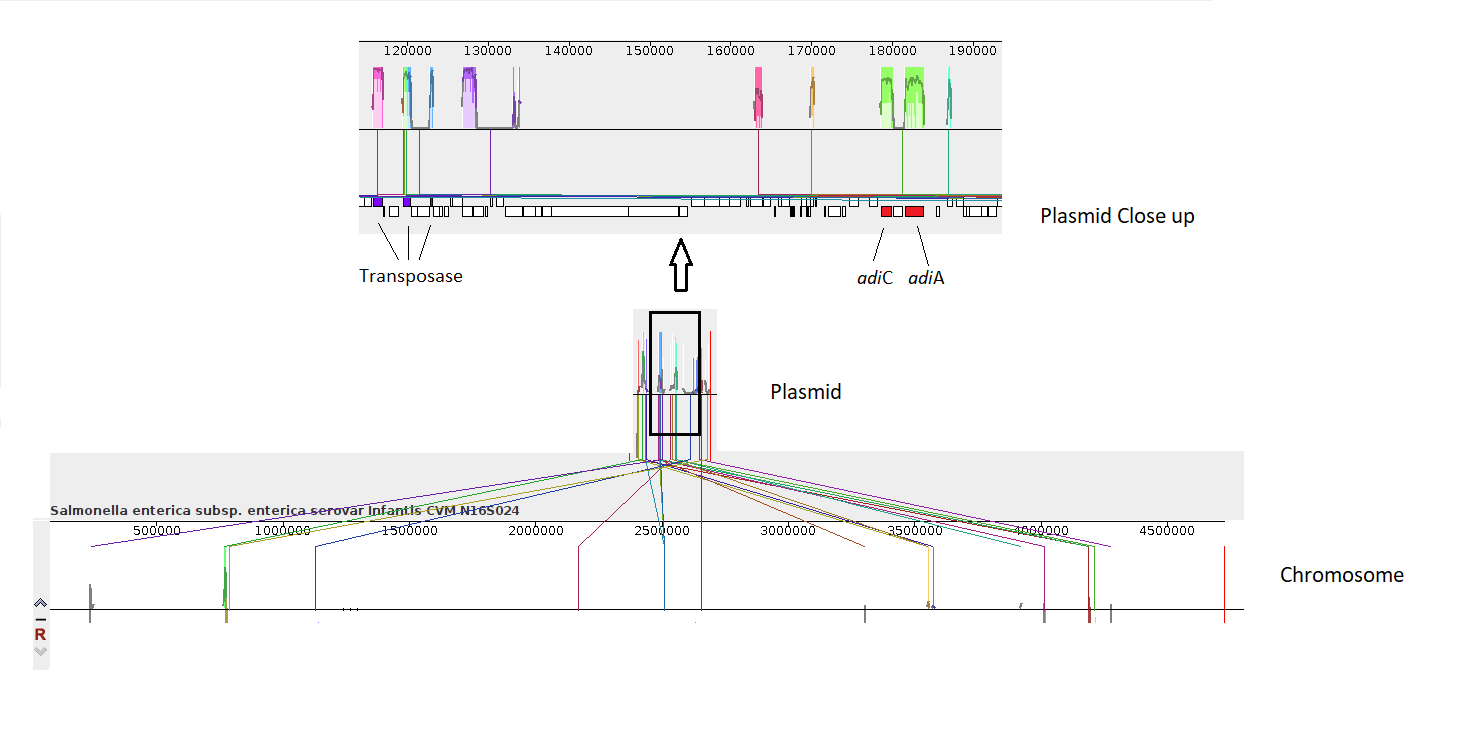

Supplement: S2 Fig — The Alignment is generated by Mauve (https://darlinglab.org/mauve/mauve.html). The color blocks show the regions with homology over 80%. (TIF) [file pone.0299354.s004.tif]

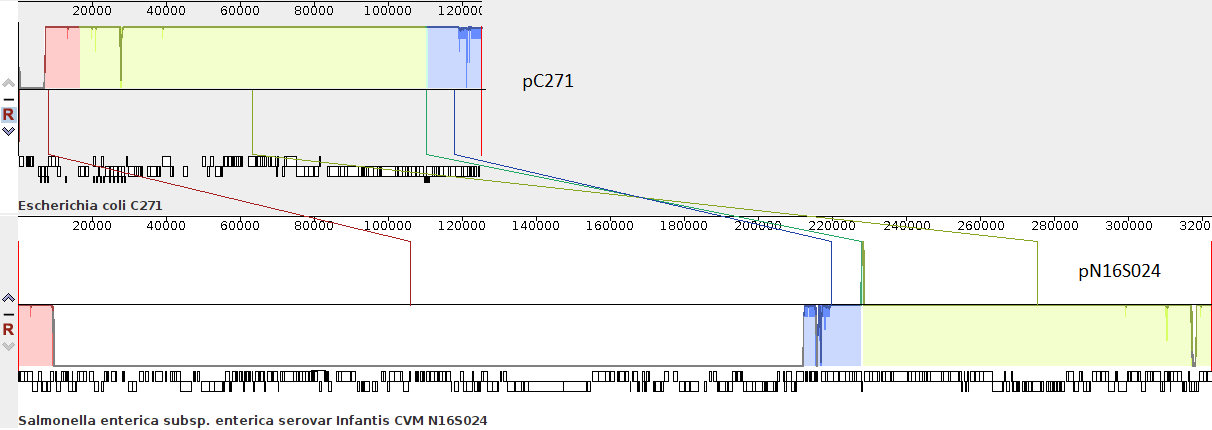

Supplement: S3 Fig — The Alignment is generated by Mauve (https://darlinglab.org/mauve/mauve.html). The color blocks show the regions with homology over 80%. (TIF) [file pone.0299354.s005.tif]
