# Supplementary material for: The spread of pESI-mediated extended-spectrum cephalosporin resistance in Salmonella serovars—Infantis, Senftenberg, and Alachua isolated from food animal sources in the United States
Source: PLoS One. 2024 Mar 14;19(3):e0299354. doi: 10.1371/journal.pone.0299354 (PMC10939224; doi:10.1371/journal.pone.0299354)
Supplement: Attachment — Submitted filename: Response_pESI.docx [file pone.0299354.s006.docx]

Journal Requirements:

When submitting your revision, we need you to address these additional requirements.

1. Please ensure that your manuscript meets PLOS ONE's style requirements, including those for file naming. The PLOS ONE style templates can be found at 
<https://journals.plos.org/plosone/s/file?id=wjVg/PLOSOne_formatting_sample_main_body.pdf> and 
<https://journals.plos.org/plosone/s/file?id=ba62/PLOSOne_formatting_sample_title_authors_affiliations.pdf>

2. We note that you have included the phrase “data not shown” in your manuscript. Unfortunately, this does not meet our data sharing requirements. PLOS does not permit references to inaccessible data. We require that authors provide all relevant data within the paper, Supporting Information files, or in an acceptable, public repository. Please add a citation to support this phrase or upload the data that corresponds with these findings to a stable repository (such as Figshare or Dryad) and provide and URLs, DOIs, or accession numbers that may be used to access these data. Or, if the data are not a core part of the research being presented in your study, we ask that you remove the phrase that refers to these data.

3. Ethics statement does not appear in the manuscript file:

Please include your full ethics statement in the ‘Methods’ section of your manuscript file. In your statement, please include the full name of the IRB or ethics committee who approved or waived your study, as well as whether or not you obtained informed written or verbal consent. If consent was waived for your study, please include this information in your statement as well.

Additional Editor Comments:

Although is an original article, major changes are required .Please try to meet the expert remarks , where possible, including a point by point reply to their comments . Look also carefully since some minor flaws are scattered throughout the text. Finally try to follow the guidelines required for article submission


Reviewers' comments:

Reviewer's Responses to Questions

**Comments to the Author**

1. Is the manuscript technically sound, and do the data support the conclusions?

The manuscript must describe a technically sound piece of scientific research with data that supports the conclusions. Experiments must have been conducted rigorously, with appropriate controls, replication, and sample sizes. The conclusions must be drawn appropriately based on the data presented.

Reviewer #1: No

Reviewer #2: Partly

2. Has the statistical analysis been performed appropriately and rigorously?

Reviewer #1: I Don't Know

Reviewer #2: No

3. Have the authors made all data underlying the findings in their manuscript fully available?

The [PLOS Data policy](http://www.plosone.org/static/policies.action#sharing) requires authors to make all data underlying the findings described in their manuscript fully available without restriction, with rare exception (please refer to the Data Availability Statement in the manuscript PDF file). The data should be provided as part of the manuscript or its supporting information, or deposited to a public repository. For example, in addition to summary statistics, the data points behind means, medians and variance measures should be available. If there are restrictions on publicly sharing data—e.g. participant privacy or use of data from a third party—those must be specified.

Reviewer #1: No

Reviewer #2: No

4. Is the manuscript presented in an intelligible fashion and written in standard English?

PLOS ONE does not copyedit accepted manuscripts, so the language in submitted articles must be clear, correct, and unambiguous. Any typographical or grammatical errors should be corrected at revision, so please note any specific errors here.

Reviewer #1: Yes

Reviewer #2: Yes

5. Review Comments to the Author

Please use the space provided to explain your answers to the questions above. You may also include additional comments for the author, including concerns about dual publication, research ethics, or publication ethics. (Please upload your review as an attachment if it exceeds 20,000 characters)

Reviewer #1: Consider suggestions to clarify this manuscript.

Line 129 authors state the Table 1 shows that each cluster carried a unique blaCTX-M allele.
But blaCTX-M-65, blaCTX-M-15, blaCTX-M-55, blaCTX-M-14, are each found in multiple clusters directly contradicting their observation and statement.

“five of the six clusters with pESI plasmid” is added on the line.

Line 374 and earlier, investigators state Fig 1A showed that the pESI plasmid spreads with clonal expansion but there are many crossovers and disagreements between the two trees that are typically interpreted as horizontal transfer and or rearrangements. So how do the authors explain this contradiction in their interpretation? Line 407, The authors seem to contradict themselves with this statement, The evolution of the plasmid does not seem to be a linear process as shown in Fig 1A. which I agree with.

In the results section, we further explain why Figure 1A shows that the pESI spreads by clonal expansion. (Lines 160-163).

Figure 1 is unreadable the authors should present a figure where the reviewer can read the isolate name on each terminal branch.

We generated a new figure 1 as below. As you can tell, it is not readable at all. We have decided to keep the same figure – however, all metadata such as location and year are indicated with color block. If anyone wants to regenerate the figure, he/she can download all the SRA based on S1 table.

Figures 5, 6 the investigators should label all terminal branches with unique isolate names.

Both figures are modified.

Data reproducibility, the authors should list all unique NCBI SRA genome ids for each isolate presented in their figures. These ids should match terminal identifications listed on their figures.

Figures 1, 5, 6 now include SRA numbers.

Reviewer #2: General Comments
S. Infantis harbouring pESI is a matter of great concern because this megaplasmid has been found to carry several (from 3 to 6) antimicrobial resistance genes, resistance genes to heavy metals and disinfectants and also other virulence factors enhancing the fitness of S. Infantis.
In this manuscript entitled “The spread of pESI…”, the authors aimed to i) understand the evolution of pESI harbouring blaCTX-M-65 by comparing using SNPs analysis all the S. Infantis sequences likely carrying pESI plasmids that were available at NBCI’s PD database at the time of the study; ii) investigate the genetic changes of the American variant of pESI-like plasmid in ESI clones . Moreover and importantly, they state they have detected other pESI-like-positive serovars beside S. Infantis, pointing out that pESI(like) spread could be associated to horizontal transfer events other than clonal expansion, as previously demonstrated.
However, some aspects about the methods used and the results obtained need to be further clarified and the discussions ensued around them, whenever necessary, should be moderated. In some cases, similar findings described in this manuscript have been already reported in recent published papers, which neither have been cited in the main text nor are present in the reference list provided by the Authors. These papers should be properly cited, as the novelties and main findings of this study should be discussed in comparison with what has been previously published (see specific comments).

Specific Comments:
- The Authors should acknowledge a possible bias in the selection of data/genomes. From Materials & Methods, only MDR S. Infantis with blaCTX-M-65 were investigated. Otherwise, the reader could understand that 99 % of the S. Infantis in the Americas were pESI-positive, which would be worrisome.

Addressed in the first paragraph of discussion.

- Along the manuscript, authors used terminology like “evolution” or “recent common ancestor”. It is necessary to know how the authors include the isolation time by using the FastTree phylogeny tool. Then in any case this represents a bias when dealing with “evolution” and “ancestry”, as the Authors have included in the study only isolates with plasmids carrying resistance to third-fourth generation cephalosporins, which is a feature that only a subset of isolates around the world do possess.

The Isolation time is from metadata in the pathogen detection database.

The query method indeed included *bla*CTX-M gene. However, it assumed that at least some isolates in the cluster(s) carried the *bla*CTX-M gene. When analysis is done, all isolates with pESI (all isolates before 2020 carried pESI plasmid, as paper stated) are included – many of them don’t carry the *bla*CTX-M gene. The search strategy is explained further in the first paragraph under discussion.

We also clarify that not all isolates within the cluster harbored the resistance genes in our search (Fig 4).

- In any case, from the data presented, analysed and discussed, the title of the manuscript should be rephrased, in order to avoid overstatements. Here is a proposal: “The spread of pESI-mediated extended-spectrum cephalosporin resistance in Salmonella Serovars - Infantis, Senftenberg, and Alachua isolated from food animal sources in the United States.” Most importantly, the title should reflect certainty on the key feature “pESI-mediated”, since (see also below) in many cases the Authors do not bring data demonstrating that all ESC resistance genes (CTX-M genes) in all serovars/isolates considered are harboured by such pESI plasmids. Otherwise, the title should be “The spread of pESI in ESBL-producing Salmonella Serovars - Infantis, Senftenberg, and Alachua isolated from food animal sources in the United States”.

Thanks for the suggestion. I have taken the suggestion and changed the title to “The spread of pESI-mediated extended-spectrum cephalosporin resistance in Salmonella Serovars – Infantis, Senftenberg, and Alachua”.

- Lines 75-76: “Our previous work and other studies have shown that pESI are transmitted mainly through clonal expansion (5, 9, 13)”. References 5 and 9 are not correctly cited here. Authors in Alba et al., 2020 reported that the pESI-like success and spread could be the result of a mixed effect of clonal expansion and horizontal transfer: “…once the megaplasmid is acquired by S. Infantis, it would likely quickly spread in the local S. Infantis population because of the presence of the specific plasmid-borne genes, which enhance the colonization, virulence and fitness behaviour of the strain…These genetic elements play a central role in bacterial adaptability in response to stress conditions and in the maintenance of plasmids or genomic islands [43], also promoting the ecological success of certain clones, such as the pESI- like- positive- ESBL- producing S. Infantis clone..”. Please rephrase your sentence, accordingly.

The sentence has been rephrased and citation has been corrected (Line74).

- Line 86: the presence of pESI-like in S. Senftenberg from US samples has been previously reported in a large-scale genomic analysis conducted on S. Infantis sequences retrieved from NCBI by dos Santos et al., 2022 (<https://www.sciencedirect.com/science/article/pii/S0740002022001368?via%3Dihub>). In particular, they have found pESI markers in four different serovars: S. Muenchen, S. Schwarzengrund, S. Agona and S. Senftenberg. This paper should be properly cited and included in the discussion. Moreover, to the best of the Reviewer's knowledge the present study represents the first documented report of the presence of pESI in S. Alachua. In this case, the novelty may be highlighted in the main text.

Thank you for this reference. The paper is now referenced on Line 88 and again in the first paragraph of the discussion.

- Line 168-172: In this paragraph and in the figure 1, the location of blaCTX-M-15 in the genome has not been clarified. The authors should check and report the location of blaCTX-M-15 gene. To the best of the reviewer’s knowledge, it has not been described any pESI harbouring bla-CTX-M-15, yet.
- In general, and most importantly (see above) they should clarify and report the location of the blaCTX-M genes in all the studied pESI-like plasmids (in some cases it has been demonstrated in previous studies and for specific serovars, but not in all pESI (like) sequences).

Although it is highly likely that blaCTX-M-15 is located on the pESI plasmid, we could not check its location since the strain with such gene is not in our possession to close its genome.

A paragraph (Line 376-380) is added to address the second half of the comment above.

- Figure 4. Additional information on how the prevalence of AMR genes for each genetic region has been calculated is missing. For example, how the normalization was done and how the authors decide from short-reads sequencing data which genes were in those regions…

More details are added to the figure legend.

- Line 301: How have the authors calculated the timing? How about the mutation rate? This should be clearly declared in the M&M section.

The sentence is reworded to include our rationale for why we believe the transfer occurred around 2020.

- Line 323: the rearrangements and structural variation in the different pESI-like plasmid variants (blaCTX-M-65, blaCTX-M-1-positive and blaCTX-M negative ones) were already demonstrated in Alba et al., 2023 (<https://academic.oup.com/femsle/article/doi/10.1093/femsle/fnad014/7049104?login=false>) . The authors should include this paper in the references. Do their findings confirm what has been reported in this recent publication?

Thank you for this reference. It is cited in discussion (Line 401) now.

- Line 345: Please cite Alba et al., 2020 (9) together with Franco et al., 2015 (5) for the ESI-CTX-M-1 cluster (in this case the Authors of the submitted manuscript refer to the bacterial host, not to the plasmid, if I am correct….).

Thank you for this suggestion. Both papers are cited (Line 358-9).

- Line 346: Although I agree with the Authors that S. Infantis clusters harbouring blaCTX-M-14 have not been previously reported, pESI harbouring blaCTX-M-14 has been previously described in Bogomazova et al., 2020 and Egorova et al., 2023 (<https://www.mdpi.com/2076-2607/11/2/347>). This latter paper should be properly cited and included in the reference list.

Both papers are cited now (Line 361). Thank you for the suggestion!

- Lines 347-349 “Our analysis…nearby countries”. This sentence concerning the “likely” origin of the ESI CTX-M-65 clone is quite speculative, also considering the available literature and should be deleted. Otherwise, the Authors should bring adequate data.

We removed the specific country names (Line 372). We feel that the Brown reference provided is sufficient to support the statement.

- Line 381: this was stated by McMillan et al. 2022: the authors should cite the paper here.

Line 413 in clean version. It is not clear why we are being asked to cite McMillan for discussing the appearance of horizontal transfer events in the turkey processing environment. That is not a feature of the McMillan paper.

- Line 399 – 406 : To validate this hypothesis, authors should verify if these genes were present or absent in the chromosome of the isolates harbouring pESI with these genes. If the gene is already present in the chromosome, its presence within the plasmid is not so important for the fitness of S. Infantis. Moreover, in isolates harbouring blaCTX-M-1, this gene is close to blaCTX-M-1 but distant from the replicon.

A supplemental S2 Fig is added and “It might have been important that the strain can survive such environment before it acquires the plasmid through horizontal transferring” is added in Line 438.

- Line 477 and following: “SNPs were called…”. Could the authors clarify this point, explaining if they have used different methods: one for all the S. Infantis and one for the ESS-CTX-M-65 phylogeny or if bowtie2 is part of the CFSAN SNP pipeline?

Yes, bowtie2 is the part of the CFSAN SNP pipeline. We have changed ‘bowtie2’ to ‘CFSAN SNP pipeline’ (Line 512).

- Line 484 The authors should add some information about the statistics (or math in general) used to get the numbers to build the figures

More details are added under the legend of Figure 4.

- Line 486: Could the authors declare the number of isolates WGSed?

Numbers have been added (Line 522-4).

- Lines 489-492 “Three Salmonella… Senftenberg”: the authors declared they have sequenced three Salmonella isolates (including S. Senftemberg and S. Alachua) by long read-sequencing. How did they demonstrate the presence of pESI plasmids (other than the presence of pESI markers) in these other two Salmonella serovars using the long-read sequencing approach? How did they locate blaCTX-M-65 and other AMR genes on these pESI plasmids? These aspects should be clearly reported in the M&M and Results section.

The annotation method is added in line 535, with a reference added. We have added a new table (Table 2) with additional details about the isolates sequenced using long read technology. All pESI plasmids were also detailed in Fig 7, including the location of blaCTX-M-65 and other resistance genes.

- Line 498: Authors should declare the accession number of the new sequences (assembly or raw reads) obtained in this study.

This information is added into a new Table 2.

- Supplementary file S2: C140670-sal, 17031040-sal, 16023650-sal isolates should be removed from spreadsheet “ESI-CTX-M-1_PDS000032463.92”as they do not harbour blaCTX-M-1 (see Supplementary table of Alba et al., 2020). The Authors should also check if the isolate UZH-SAL-119-15 from Switzerland harbour blaCTX-M-1. I suggest to re-check the AMR gene content of all the sequences reported in the Supplementary file S2.

It seems that there are two of C140670-sal:

| C140670-sal | PDT001014877.1 | | PRJEB23728 | | aadA1,aph(3')-Ia,dfrA14,gyrA_D87G=POINT,sul1,tet(A) | |
| --- | --- | --- | --- | --- | --- | --- |
| C140670-sal | PDT001235767.1 | PRJNA771355 | | aadA1,blaCTX-M-1,dfrA14,dfrA1,gyrA_D87G=POINT,sul1,tet(A) | |  |

One with blaCTX-M-1 another without. They are under the different Bioproject. Is it likely the same name used for both isolates?

The other three did not carry blaCTX-M-1 on the table:

| 17031040-sal | PDT001014880.1 | | PRJEB23728 | | aadA1,aph(3')-Ia,blaTEM-1,dfrA14,gyrA_D87G=POINT,lnu(G),sul1,tet(A) | |
| --- | --- | --- | --- | --- | --- | --- |
| 16023650-sal | PDT001014885.1 | | PRJEB23728 | | aadA1,blaTEM-1,dfrA14,gyrA_D87G=POINT,lnu(G),sul1,tet(A) | |
| UZH-SAL-119-15 | PDT000219937.1 | PRJNA378379 | | aadA1,aph(3')-Ia,dfrA14,gyrA_D87G=POINT,sul1,tet(A) | |  |

Therefore, nothing changed there.

Thanks for checking in detail!

6. PLOS authors have the option to publish the peer review history of their article ([what does this mean?](https://journals.plos.org/plosone/s/editorial-and-peer-review-process#loc-peer-review-history)). If published, this will include your full peer review and any attached files.


If you choose “no”, your identity will remain anonymous but your review may still be made public.


**Do you want your identity to be public for this peer review?** For information about this choice, including consent withdrawal, please see our [Privacy Policy](https://www.plos.org/privacy-policy).

Reviewer #1: No

Reviewer #2: No

[NOTE: If reviewer comments were submitted as an attachment file, they will be attached to this email and accessible via the submission site. Please log into your account, locate the manuscript record, and check for the action link "View Attachments". If this link does not appear, there are no attachment files.]

While revising your submission, please upload your figure files to the Preflight Analysis and Conversion Engine (PACE) digital diagnostic tool, <https://pacev2.apexcovantage.com/>. PACE helps ensure that figures meet PLOS requirements. To use PACE, you must first register as a user. Registration is free. Then, login and navigate to the UPLOAD tab, where you will find detailed instructions on how to use the tool. If you encounter any issues or have any questions when using PACE, please email PLOS at [figures@plos.org](mailto:figures@plos.org). Please note that Supporting Information files do not need this step.
