# Supplementary material for: The spread of pESI-mediated extended-spectrum cephalosporin resistance in Salmonella serovars—Infantis, Senftenberg, and Alachua isolated from food animal sources in the United States
Source: PLoS One. 2024 Mar 14;19(3):e0299354. doi: 10.1371/journal.pone.0299354 (PMC10939224; doi:10.1371/journal.pone.0299354)
Supplement: Attachment — Submitted filename: Response110123.docx [file pone.0299354.s007.docx]

-The bias of the isolate/genome selection has now been declared in the discussion part, but it has not been considered when formulating the conclusions. For example, in line 169-171 (tracked changes version), the Authors have stated: “If the evolution of the plasmids was not affected by the host, it would not form clades that are similar in structure…”. The authors should consider whether the cluster topology would change if other S. infantis isolates (CTX-M-neg or pESI neg) were included. This is important, as this phenomenon (agreement of chromosomal SNP tree and plasmid SNP tree) has not been observed in the previous literature on the topic (Alba et al., 2020).

The cluster ESI-CTX-M-65 (PDS000089910.296) was identified by NCBI clustering method. All isolates are withing 50 SNPs difference from each other as stated between line 101 to 104. As we have stated (line 151-152), all *Salmonella* infantis strains before 2016 are included in tree. We were not biased in our selection, there simply are no *Salmonella* infantis strains before 2016 without plasmid (S1_table). In fact, all except one isolate in the clusters have the replicon *Inc*FIB(pN55291). The one without this replicon marker had all other six markers. This shows that every single strain had at least some portion of the plasmid. This is detailed between line 197 to line 206.

Therefore, we conclude that there is no bias.

-The statement that ESI-CTX-M-65 derived from ESI-CTX-M15 is an overstatement because there is no evidence of it in the Authors’ experiments. Maybe, from the fig 1 (without knowing the number of SNPs differences or the length of the branch) you can say/infer that the chromosomal content is very similar, but not the plasmid tree. In their own figure, the Authors can observe how huge the branch length on the plasmid tree is. If they write on the manuscript that ESI CTX-M-65 derived from ESI CTX-M-15, the general educated reader could think that the plasmid had evolved from a CTX-M-15-positive pESI, but there is no evidence of it from this study. From their data, I have interpreted that the clone (chromosome) is similar (one would need to know the SNPs difference) but not the plasmid sequence. Maybe the CTX-M-15 had pESI without blactx-m-15 and blactx-m-15 could either be located in other plasmids or this isolate could harbor only the IncI half part of the pESI plasmid.
And, in any case, and most importantly, if S. Infantis did not harbour pESI, it should not be named ESI.

We did not name any cluster as “ESI-CTX-M-15” as the strains with *bla*CTX-M-15 did not form a separate cluster. The cluster “ESI-CTX-M-65” includes ESI-CTX-M-15 since majority of the isolates carried blaCTX-M-65. Only early isolates from human in Peru carried *bla*CTX-M-15 (Line 170-176). The reviewer is right that *bla*CTX-M-15 could be on chromosome. We have never stated its location. We only concluded that the strains with blaCTX-M-65 came from the strains with *bla*CTX-M-15 solely based on the phylogenetic tree.

Yes, some of the branch length of plasmid tree is longer than those on chromosomal tree as reviewer observed. We have mentioned the plasmid evolved in a “step wise” fashion (line 166-167), most likely resulting from recombination, as discussed (line 440-442).

The plasmid tree used SNPs on the homologous region to predict the evolutionary path, and it is mostly consistent with chromosomal tree. I do think it indicates the plasmid evolves with its host chromosome.

We define ESI as Emerging *Salmonella* Infantis (Line 50). We don’t see the problem with naming the clusters as “ESI-XXX”.

-The Authors answered that the reconstruction of timing of pESI introduction and evolution was based on S. infantis isolation time from metadata. This is not enough information to talk about “evolution”. The isolation time let one speculate on possible scenario/s, however it does not provide a “probabilistic evolutionary model”, that need to be calculated to predict when pESI was introduced and then evolved in the S. Infantis population described in this study. The Authors should not refer and use terms like “evolution” throughout the text, unless they provide evidence on how it was calculated, in particular when they refer to pESI plasmid. This also represents a limitation of this study that should be clearly underlined in the discussion section.

“Evolution” is a broad term that is being used by scientist to describe the change in inheritable characteristic of biological populations over successive generations. Natural selection and generic drift contribute to evolution process. We never used isolation time of a strain alone to describe the “evolution”, instead, we combined it with phylogenetic tree to describe the “evolution”. We never provided a “probabilistic evolutionary model”, which I agree that it needs more analysis to calculate.

Response to Minor comments:

-The isolate C140670-sal to which I refer was included in the study Alba et al., 2020 (PRJEB23728) where its sequence was originally included.

Got it. Thanks!

-Isolates 17031040-sal, 16023650-sal, UZH-SAL-119-15 (CTX-M negative) were included in a spreadsheet named “ESI-CTX-M-1_PDS000032463.92”. Could the authors clarify what kind of sequences are included in the different spreadsheets of Supplementary File S2? What is the common feature among these groups of sequences? In case, I suggest to rename the different spreadsheets, accordingly.

All information of the isolates in this paper were downloaded from NCBI pathogen detection browser database, including assemblies, SRA, AMR and clusters names. We only added the cluster names we used in this paper as pre-fix in S1 and S2_table. We did not modify any data since the information are from public record. The legends and spreadsheets for S1 and S2 table are modified a little and hopefully it is clearer now.
