# Supplementary material for: The spread of pESI-mediated extended-spectrum cephalosporin resistance in Salmonella serovars—Infantis, Senftenberg, and Alachua isolated from food animal sources in the United States
Source: PLoS One. 2024 Mar 14;19(3):e0299354. doi: 10.1371/journal.pone.0299354 (PMC10939224; doi:10.1371/journal.pone.0299354)
Supplement: Attachment — Submitted filename: Response_02012024.docx [file pone.0299354.s008.docx]

Journal Requirements:

Please review your reference list to ensure that it is complete and correct. If you have cited papers that have been retracted, please include the rationale for doing so in the manuscript text, or remove these references and replace them with relevant current references. Any changes to the reference list should be mentioned in the rebuttal letter that accompanies your revised manuscript. If you need to cite a retracted article, indicate the article’s retracted status in the References list and also include a citation and full reference for the retraction notice.

Additional Editor Comments:

Dear Authors,

You have definitely improved the manuscript, addressing most of comments both in the revised copy and in point-by-point response letters for the reviewers . However some minor changes are still required for pubblication. Please consider the following reviewer's comments

The authors have compiled a well-written manuscript that adds to the narrative of the spread of pESI-mediated extended-spectrum cephalosporin resistance in Salmonella serovars - Infantis, Senftenberg, and Alachua in the United States. However, from the narrative, it is not totally clear to me at the beginning if it was an actual study where isolates were analyzed or if sequences were retrieved from NCBI for the analysis. It is a bit confusing
At a certain point, it reads as if data were retrieved from the database and at the method sections it reads as if you conducted the actual test. Please clarify line 520. Though some isolates were received from NARMs it is not clear at the beginning of the manuscript.

One sentence was added to clarify the source of data: “The SRAs were submitted to NCBI pathogen detection database and were downloaded subsequentially for analysis, along with other SRAs deposit in PD database by other institutes.”

Line 72-73 “Additionally, some Salmonella Infantis strains with pESI-like plasmid have been found carrying a colistin-resistance gene (mcr-1) (17, albeit not on the pESI-like plasmid, further limiting treatment options”. Could you please clarify this statement as the understanding is not clear?

Changed it to “Additionally, some *Salmonella* Infantis strains with pESI-like plasmid have been found carrying a colistin-resistance gene (*mcr*-1) (17, 18), albeit on a IncX4, further limiting treatment options, as colistin is considered as a last resort for infection. “

Line 92 in this study is this a study or review

We used “pESI” to refer to all “pESI” and “pESI-like” plasmids used by other papers in this study.

Line 128-134 Only 13 out of 5765 (0.2%) blaCTX-M alleles are blaCTX-M-15, 5687 (98.6%) are blaCTX-M-65, the remaining 65 are unnamed blaCTX-M alleles (S1Table). It seems the numbers do not add up. Can you explain where these numbers are coming from?

13 + 5687 + 65 = 5765. I think it is correct.

Line 192 I am not sure where 8935 is coming from it is not on the table “143 of the 8,935 isolates in this cluster with”

All numbers are rechecked and recalculated in this paragraph. Thanks for checking.

Line207 can you specifics how many strains from NARM was used

“41,280 strains in total” is added. Thanks for your suggestion.

Line 446 E. coli should be italicized

Corrected. Thanks!

Line 503. “Isolates from other countries were selected randomly based on location, source, and year” This is not clear to me. kindly indicate where are the isolates coming from and why that selections.

I see your point. “randomly” is deleted now. It is changed to “to represent different locations, sources, and years.” Thank you!

[Note: HTML markup is below. Please do not edit.]

Reviewers' comments:

Reviewer's Responses to Questions

**Comments to the Author**

1. If the authors have adequately addressed your comments raised in a previous round of review and you feel that this manuscript is now acceptable for publication, you may indicate that here to bypass the “Comments to the Author” section, enter your conflict of interest statement in the “Confidential to Editor” section, and submit your "Accept" recommendation.

Reviewer #2: (No Response)

Reviewer #3: All comments have been addressed

Reviewer #4: All comments have been addressed

2. Is the manuscript technically sound, and do the data support the conclusions?

The manuscript must describe a technically sound piece of scientific research with data that supports the conclusions. Experiments must have been conducted rigorously, with appropriate controls, replication, and sample sizes. The conclusions must be drawn appropriately based on the data presented.

Reviewer #2: Partly

Reviewer #3: Yes

Reviewer #4: Yes

3. Has the statistical analysis been performed appropriately and rigorously?

Reviewer #2: No

Reviewer #3: Yes

Reviewer #4: Yes

4. Have the authors made all data underlying the findings in their manuscript fully available?

The [PLOS Data policy](http://www.plosone.org/static/policies.action#sharing) requires authors to make all data underlying the findings described in their manuscript fully available without restriction, with rare exception (please refer to the Data Availability Statement in the manuscript PDF file). The data should be provided as part of the manuscript or its supporting information, or deposited to a public repository. For example, in addition to summary statistics, the data points behind means, medians and variance measures should be available. If there are restrictions on publicly sharing data—e.g. participant privacy or use of data from a third party—those must be specified.

Reviewer #2: Yes

Reviewer #3: Yes

Reviewer #4: Yes

5. Is the manuscript presented in an intelligible fashion and written in standard English?

PLOS ONE does not copyedit accepted manuscripts, so the language in submitted articles must be clear, correct, and unambiguous. Any typographical or grammatical errors should be corrected at revision, so please note any specific errors here.

Reviewer #2: Yes

Reviewer #3: Yes

Reviewer #4: Yes

6. Review Comments to the Author

Please use the space provided to explain your answers to the questions above. You may also include additional comments for the author, including concerns about dual publication, research ethics, or publication ethics. (Please upload your review as an attachment if it exceeds 20,000 characters)

Reviewer #2: The Authors have not addressed major comments on the R2 version of the manuscript, so the study limits and overstatements i have observed are still present in the main text.

I believe the major concern has been answered.

Reviewer #3: The study of pESI expansion and evolution would help public health scientists to understand, monitor and contain its spread through targeted mitigation strategies.
L94 and 98, PD need full spelling when first appear.

It is corrected. Thanks for checking.

L99, NARMS need full spelling.

Corrected. Thanks for checking.

L118 six clusters carried pESI plasmid, why to choose this? Need proofs.

We used seven markers to check all eight clusters (Listed in table 1) and only strains in six of them carried the pESI, which is the aim of this study.

L123 2023 year?

“2023” is added. Thanks for checking.

L315 add USA

“USA” is added. Thanks for suggestion.

Reviewer #4: The authors have compiled a well-written manuscript that adds to the narrative of the spread of pESI-mediated extended-spectrum cephalosporin resistance in Salmonella serovars - Infantis, Senftenberg, and Alachua in the United States. However, from the narrative, it is not totally clear to me at the beginning if it was an actual study where isolates were analyzed or if sequences were retrieved from NCBI for the analysis. It is a bit confusing
At a certain point, it reads as if data were retrieved from the database and at the method sections it reads as if you conducted the actual test. Please clarify line 520. Though some isolates were received from NARMs it is not clear at the beginning of the manuscript.

One sentence is added to clarify the source of data: “The SRAs were submitted to NCBI pathogen detection database and were subsequently downloaded for analysis, along with other SRAs deposit in PD database by other institutes.”

Line 72-73 “Additionally, some Salmonella Infantis strains with pESI-like plasmid have been found carrying a colistin-resistance gene (mcr-1) (17, albeit not on the pESI-like plasmid, further limiting treatment options”. Could you please clarify this statement as the understanding is not clear?

Changed it to “Additionally, some *Salmonella* Infantis strains with pESI-like plasmid have been found carrying a colistin-resistance gene (*mcr*-1) (17, 18), albeit on the IncX4, further limiting treatment options, as colistin is considered as a last resort for infection. “

Line 92 in this study is this a study or review

We used “pESI” to refer to all “pESI” and “pESI-like” plasmids used by other papers in this study.

Line 128-134 Only 13 out of 5765 (0.2%) blaCTX-M alleles are blaCTX-M-15, 5687 (98.6%) are blaCTX-M-65, the remaining 65 are unnamed blaCTX-M alleles (S1Table). It seems the numbers do not add up. Can you explain where these numbers are coming from?

13 + 5687 + 65 = 5765. Did I miss anything here?

Line 192 I am not sure where 8935 is coming from it is not on the table “143 of the 8,935 isolates in this cluster with”

All numbers are rechecked and recalculated in this paragraph. Thanks for checking.

Line207 can you specifics how many strains from NARM was used

“41280 in total” is added. Thanks for your suggestion.

Line 446 E. coli should be italicized

Corrected. Thanks!

Line 503. “Isolates from other countries were selected randomly based on location, source, and year” This is not clear to me. kindly indicate where are the isolates coming from and why that selections.

I see your point. “randomly” is deleted now. It is changed to “to represent different locations, sources, and years.” Thank you!

7. PLOS authors have the option to publish the peer review history of their article ([what does this mean?](https://journals.plos.org/plosone/s/editorial-and-peer-review-process#loc-peer-review-history)). If published, this will include your full peer review and any attached files.


If you choose “no”, your identity will remain anonymous but your review may still be made public.


**Do you want your identity to be public for this peer review?** For information about this choice, including consent withdrawal, please see our [Privacy Policy](https://www.plos.org/privacy-policy).

Reviewer #2: No

Reviewer #3: **Yes:** Qingli Dong

Reviewer #4: No
